# A pilot investigation of condylar position and asymmetry in patients with unilateral posterior scissors-bite malocclusion based on three-dimensional reconstructive imaging technique

**DOI:** 10.1186/s12891-023-06384-z

**Published:** 2023-04-01

**Authors:** Chen-xi Li, Xin Xie, Mengjia Li, Zhong-cheng Gong, Jing Nie, Xu Liu, Sakendeke Jumatai

**Affiliations:** 1 Department of Oral and Maxillofacial Oncology & Surgery, Xinjiang Medical University Affiliated First Hospital, School / Hospital of Stomatology Xinjiang Medical University, Stomatological Research Institute of Xinjiang Uygur Autonomous Region, No.137 Liyushan South Road, Urumqi, 830054 P.R. China; 2 Hubei Province Key Laboratory of Oral and Maxillofacial Development and Regeneration, Wuhan, 430022 P.R. China; 3 Department of Orthodontics, Xinjiang Medical University Affiliated First Hospital, School / Hospital of Stomatology Xinjiang Medical University, Urumqi, 830054 P.R. China; 4 Department of Maxillofacial Surgery, Hospital of Stomatology, Key Laboratory of Dental-Maxillofacial Reconstruction and Biological Intelligence Manufacturing of Gansu Province, Faculty of Dentistry, Lanzhou University, Lanzhou, 730013 P.R. China; 5 Department of Oral and Maxillofacial Radiology, Xinjiang Medical University Affiliated First Hospital, Urumqi, 830054 China

**Keywords:** Unilateral posterior scissors bite, Condyloid process, Glenoid fossa, Three-dimensional reconstruction, Image registration

## Abstract

**Objective:**

Unilateral posterior scissors-bite (uPSB) malocclusion is common clinically. This study aimed to investigate the condylar morphological alterations and condyle-fossa relationship in patients with uPSB, through cone beam computed tomography (CBCT) and three-dimensional reconstructive imaging technique.

**Methods:**

A retrospective study was designed to comparatively analyze 95 patients with uPSB between July 2016 and December 2021. They were divided into three subgroups: 12 to 20, 21 to 30, and ≥ 31 years, according the age distribution. The morphological parameters regarding condyle, fossa, and joint space after three-dimensional reconstruction were measured and analyzed by a series of digital software. SPSS 26.0 software package was performed for statistical analysis on data sets, using paired *t*–test, one–way analysis of variance, Wilcoxon signed–rank sun test, Kruskal–Wallis *H* test, and Bonferroni correction.

**Results:**

The condylar volume (CV) of scissors-bite side was greater than that of the non-scissors-bite side (*CV*_A_ = 1740.68 ± 559.80 mm^3^ > *CV*_N_ = 1662.25 ± 524.88 mm^3^, *P* = 0.027). So was the condylar superficial area (CSA) (*CSA*_A_ = 818.71 ± 186.82 mm^2^ > *CSA*_N_ = 792.63 ± 173.44 mm^2^, *P* = 0.030), and the superior joint space (SJS) [*SJS*_A_ = 2.46 (1.61, 3.68) mm) > *SJS*_N_ = 2.01 (1.55, 2.87) mm), *P* = 0.018], and the anterior joint space (AJS) (*AJS*_A_ = 3.94 ± 1.46 mm > *AJS*_N_ = 3.57 ± 1.30 mm, *P* = 0.017). The constituent ratios of the different parts of the bilateral condyles were 23% on the posterior slope, 21% on the top, 20% on the anterior slope, 19% on the lateral slope and 17% on the medial slope, respectively.

**Conclusion:**

Due to long-term abnormal occlusion of uPSB, the pathological bite force in temporomandibular joint would cause changes in the shape of the condyle. Among them, CV, CSA, SJS and AJS had significant changes in the scissors-bite status, which has the greatest damage to the posterior slope of the condyloid process.

## Introduction

Scissors-bite, a common type of malocclusion, is characterized by linguoversion or lingual inclination of the mandibular posterior teeth or/and buccoversion or buccal inclination of the maxillary posterior teeth, resulting in reduced contact of occlusal surfaces [1]. The prevalence of scissors-bite condition in children and adolescents is 2.2 ± 3.4% and in adults is 5.0 ± 6.5%, respectively [2, 3]. Unilateral posterior scissors-bite (uPSB) occurs more frequently than bilateral PSB, resulting from organic causes, iatrogenic causes and functional growth problems [4]. Clinically, uPSB patients are often prone to unilateral and habitual mastication of healthy side. Therefore, it is reported that a series of problems such as pathological tooth flaring, drifting, and elongation, dental caries and poor periodontal hygiene are often found on the PSB side during oral examination [5]. Mechanically and geometrically based ideas on the association between dental malocclusion, *e.g.*, uPSB, and masticatory dysfunction, maxillofacial asymmetry, and temporomandibular disorders (TMD) have been one of the most controversial topics [6]. The principles of TMD treatment aim at neuromuscular relief, orthodontic and/or orthopedic correction of the craniomandibular relationship. Modalities or devices with respect to pain control, decrease or elimination of muscle dysfunction and/or internal derangements, which have been proven themselves effective in clinical practice, when based upon reasonable documentation, are considered appropriate [7].

As the center of craniomaxillofacial growth and development, condyloid process is the crucial structure that articulates with the disk of the temporomandibular joint (TMJ). It outgrows multi-directionally and has the capacity of adaptive remodeling. When the TMJ is subjected to abnormal bite force or long-term physical stress exceeds its own adaptive capacity, the condyle will undergo functional remodeling, leading to morphological changes and ultimately affecting the condylar size [8]. This adaptability is of great importance, since the condyle can be modified by persistent anomalous occlusal contact. Some studies have assessed the impact of uPSB on the muscles of masticatory apparatus [1, 9, 10], but few studies have investigated the bony changes of TMJ in individuals suffered from uPSB. This is interesting because if such individuals show bony changes of TMJ in uPSB side dissimilar to the contralateral side with normal occlusion, then the orthodontic treatment should be considered pretty necessary.

A detailed medical interview, careful check for relationship of natural occlusal plane with different anatomical landmarks, and further imaging examinations including bone scintigraphy, radiography and computed tomography (CT) may lead to the diagnosis of potential growth of the condyle, especially when the asymmetric malocclusions are observed [11, 12]. Even though bone scintigraphy is thought to be an excellent tool to assess condylar viability, it is considerably confined because of the additional cost and radiation exposure [11, 13]. On the other hand, the results of initial bone scintigraphy did not show sufficiently close associations with the prognosis of long-term degenerative joint alterations [11].

In recent years, the technology of evaluating and analyzing teeth and soft and hard tissues of TMJ in three-dimensional (3D) plane has become more and more mature [14]. Dimensional images, acquired using cone beam CT (CBCT) scanning data, are becoming increasingly popular in the clinical work and research. Although more and more biometric studies used 3D digital models and CBCT on measuring analysis were admitted, most of them were still on the basis of an old 2D fashion to assess “point-to-point” rather than “surface-to-surface” distances. The purpose of this imaging study was to observe the position and morphological changes of bilateral condyles in the articular fossa in patients with uPSB malocclusion applying CBCT and 3D reconstructive technique, so as to further evaluate the condylar asymmetry following the methodology of bilateral condyles image registration at a time.

## Materials and methods

### Study design

A cross-sectional retrospective study was designed and implemented to reasonably address the research purpose. Patients suffered with uPSB malocclusion admitted to the Temporomandibular Joint Specialist Clinic, Xinjiang Medical University Affiliated First Hospital, China, from July 2016 to December 2021 constituted the study population.

The study protocol was reviewed and approved through the Ethics Committee, Faculty of Dentistry, Xinjiang Medical University Affiliated First Hospital (approval number K202208-04, grant date 5th August 2022). Procedures in this research were completed following the standards of the Declaration of Helsinki. Written informed consent was obtained from all subjects/ legal guardians. All data generated or analyzed during this study were included in this published article.

### Sample resource

#### Inclusion criteria

Patients who met any of the following criteria were included: (i) presence of unilateral posterior scissors-bite affecting one or more teeth; (ii) unilateral posterior scissors-bite that had not been treated previously, including nonsurgical orthodontic treatment, distraction osteogenesis, prosthodontic rehabilitation, etc.; (iii) mixed dentition/ full permanent dentition (with or without molars); (iv) no history of trauma, tumor or infection in TMJ.

#### Exclusion criteria

The exclusion criteria were as follows: (i) primary and/or secondary crowding of the dentition; (ii) congenital craniomaxillofacial abnormalities (*e.g.*, isolated hypoplasia of the condyle, Treacher Collins syndrome, Goldenhar syndrome, etc.); (iii) radiographic examination showed organic lesions in the TMJ or/and any other TMJ disease; (iv) a history of neuromuscular disorders, rheumatism and other systemic diseases; (v) contraindications for CBCT examinations.

#### Patient eligibility

**Fig. 1:**
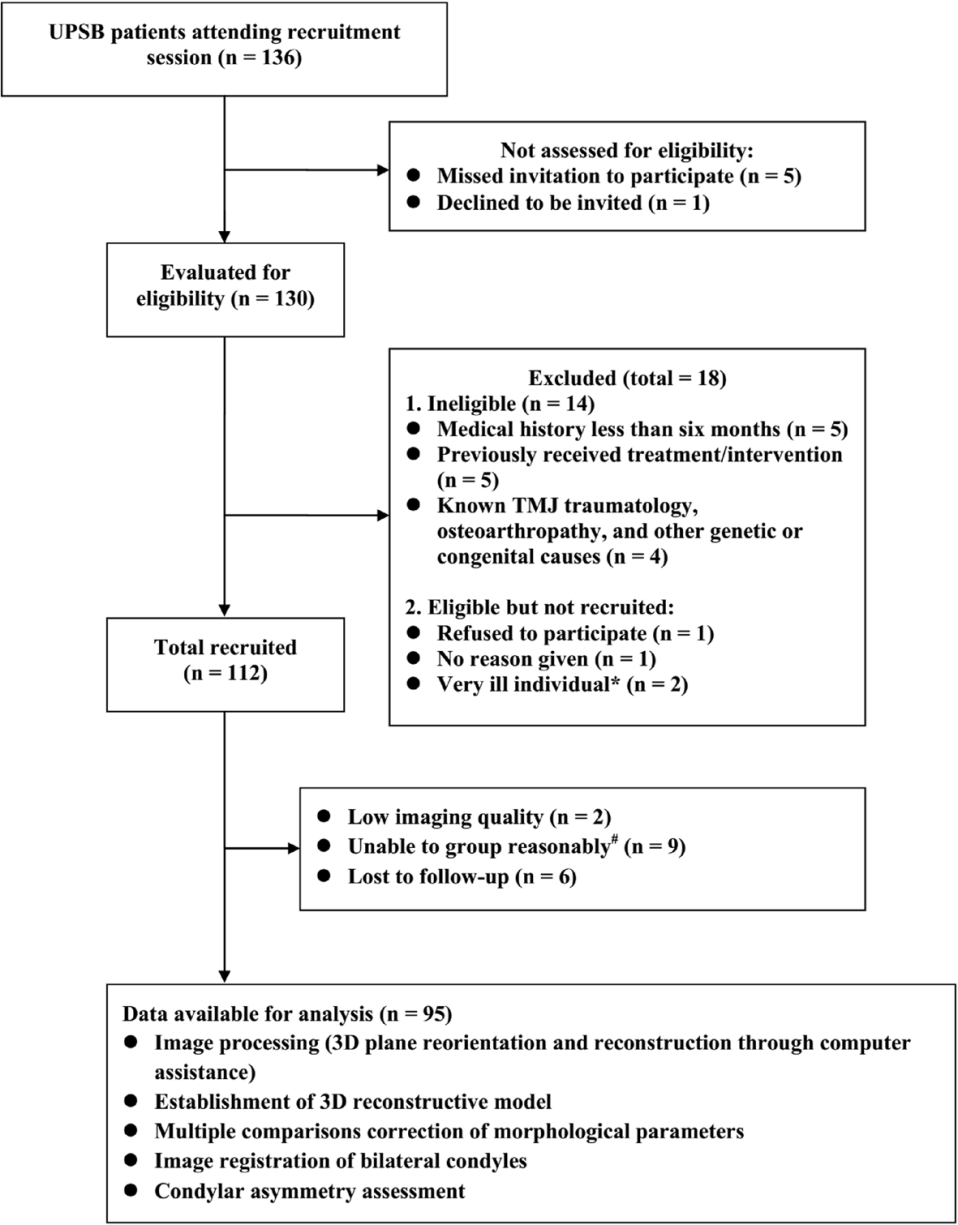
Study flowchart. *Obvious organ dysfunction or organ failure, including failing heart with level IV of cardiac function, kidney failure with estimated glomerular filtration rate (eGFR) below 60 mL/min, liver failure with aminopherase above 1,600 U/L, TBil (total bilirubin) above 171 μmol/L, with/without hepatic encephalopathy, coagulation defects with PTA (prothrombin activity) below 40%. ^#^No reconsultation data from medical record; reconsulted but no diagnosis given; all data lost

By accessing medical record files, in total 136 patients met the inclusion criteria. All patients accepted to take part in the investigation, but the imaging materials of two potential participants were inadequate/lost to 3D reconstruct, and 9 cases suffering from bilateral PSB were excluded since they failed to meet the self-control design which required a uPSB side and a normal occlusion side from one patient. Finally, 95 patients, with uPSB, were enrolled for the present imaging study. A flow diagram describing the subjects’ enrollment as well as the working plan is given in Fig. 1, according to the STROBE (Strengthening the Reporting of Observational Studies in Epidemiology) statement [15].

In keeping with a self-controlled case series design, from one patient, the uPSB side was set as cases (*n* = 95) and the normal side was set as controls (*n* = 95). In addition, the included subjects were also divided into three subgroups according to different ages: respectively, 12 ~ 20 years, 21 ~ 30 years, ≥ 31 years.

### CBCT image acquisition

A high-resolution CBCT scanner equipped with a head positioner that supplemented by cursor positioning system (Galileos®COMFORT^PLUS^, Sirona Dental Systems GmbH) was utilized for the bilateral TMJs’ examination. All participants who underwent the spatial, volumetric CBCT scanning were under uniform conditions and without sedatives and in keeping with the standard posture [16]. The thickness layer of the scanning process was required as 0.15 mm. The technical parameters were as follows: effective tube current = 7 mA, tube voltage = 85 kV peak, matrix = 512 × 512, field of view (FOV) = 20 cm × 19 cm, revolution speed = 1 r/S (rotation/second), total scanning time = 15 s. The protocol of CBCT Sirona 3D unit was 0.625 mm for reconstructed slice thickness, and 0.5 mm for reconstructive interval, respectively. More detailed guidelines of CBCT scanning was described in our previous work (https://pubmed.ncbi.nlm.nih.gov/36096796/). All CBCT images were analyzed by two clinicians (a radiologist Dr. S. Jumatai and an oral and maxillofacial specialist Prof. Dr. Dr. Z. Gong).

### Processing of imaging materials and data measurements

**Fig. 2:**
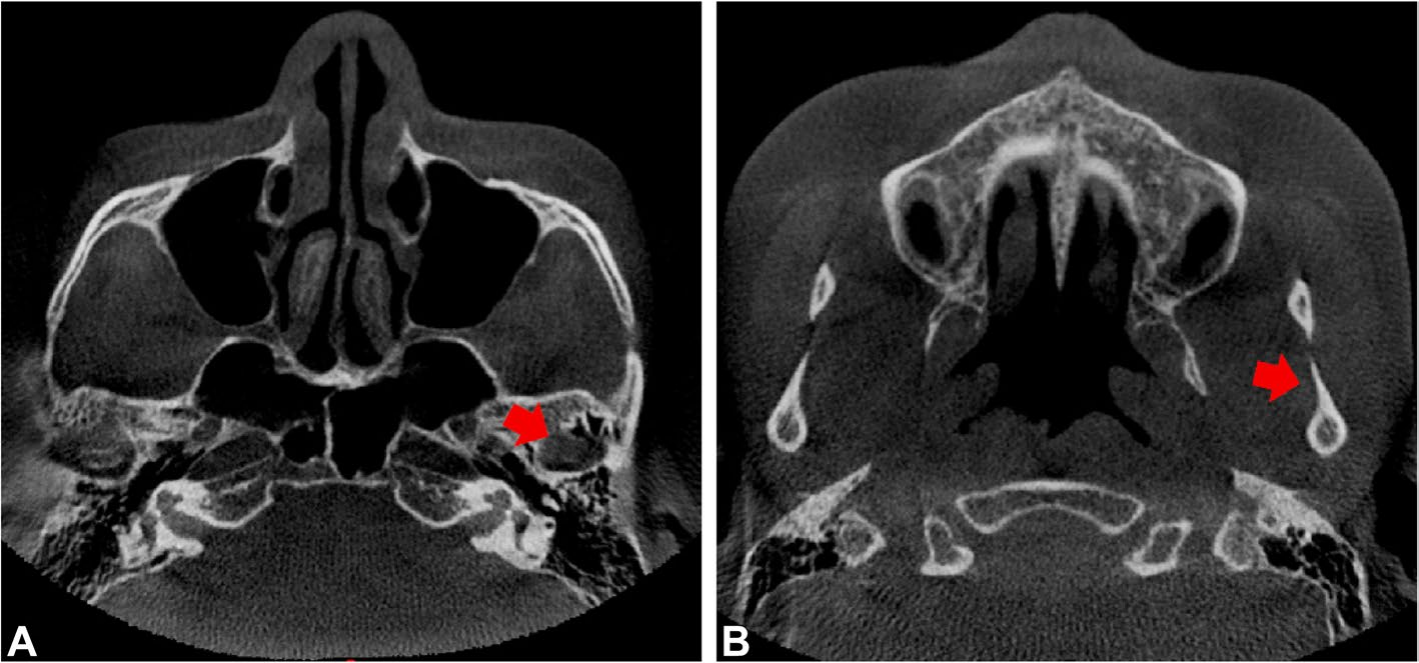
Determination of the condylar boundary in the coronal plane. **A** Appearance of the first high-density shadow defined as the top of the condyle. **B** First separation of the coracoid process and condyle is regarded as the bottom of the condyle

**Fig. 3:**
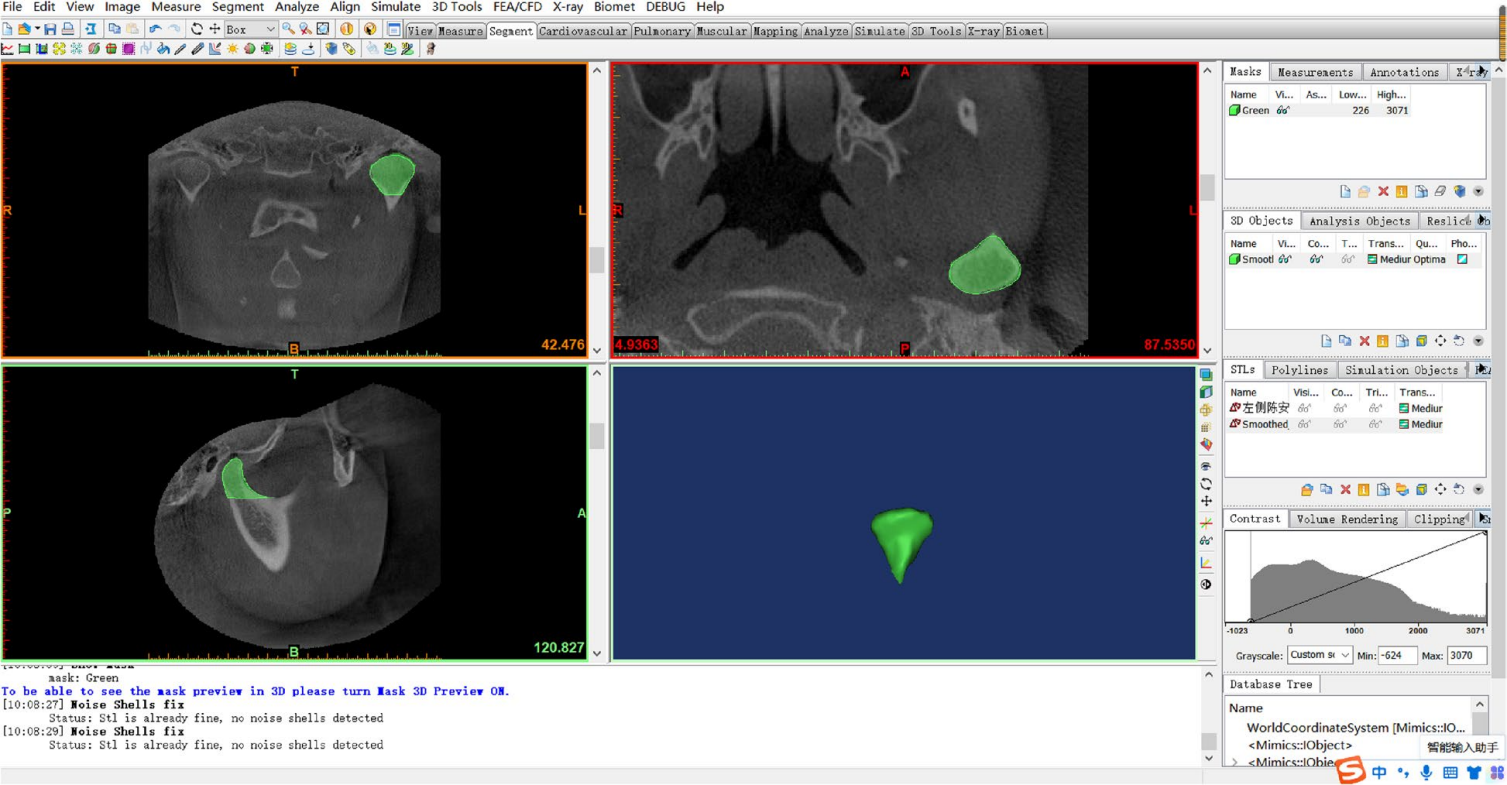
Reconstruction of the condyloid process. Use of the Multiple Slice Edit plugin to select the condylar range within its boundary at the coronal, axial, and sagittal levels. Using Smooth and Wrap instruction to refine the contour. The 3D reconstructed condyle is modeled

**Fig. 4:**
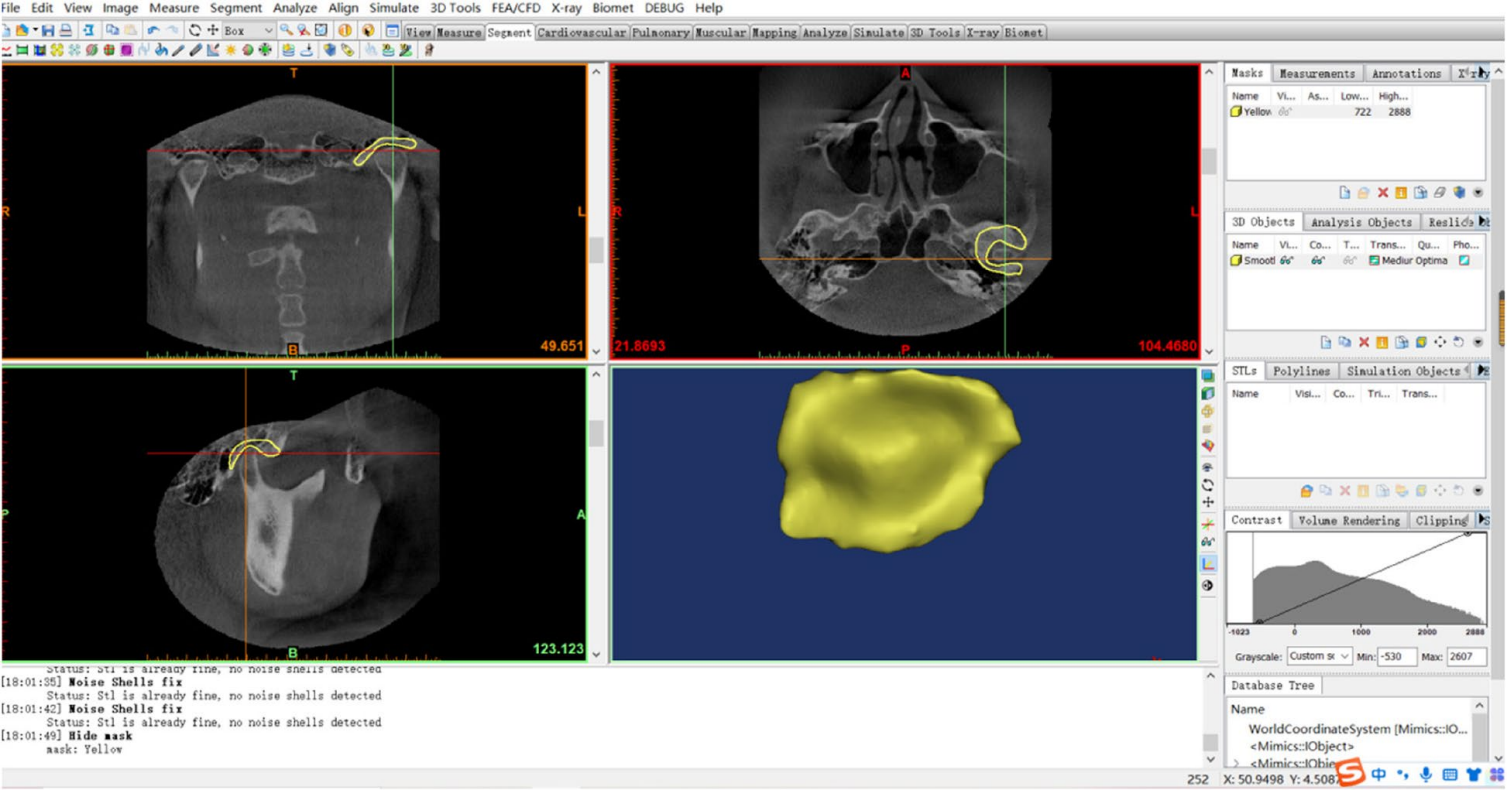
Reconstruction of the articular fossa. Use of the Crop Mask command to select the region of articular fossa. Use of the Multiple Slice Edit plugin to erase the partial condyle at the coronal, axial, and sagittal levels. Further removal of the condyle via the Region Growing command. Use of the Smooth and Wrap command to refine the contour. 3D reconstructed fossa is modeled

All data generated or analyzed during this study were included in this published article. CBCT data were generated as DICOM format which were exported to the processing and analyzing system workstation (Sidexis XG Digital Radiography, Sirona Dental Systems GmbH) and imported to Mimics for Windows (version 19.0, Mimics software, Materialise) for 3D plane reorientation and reconstruction. By reorienting every plane, the 3D parameterized modeling was performed under a grayscale thresholds of 226─3071 Hounsfield units, to determine the condyle boundary (Fig. 2), accomplish 3D reconstruction of the condyle (Fig. 3), and the glenoid fossa (Fig. 4).

The following 12 representative morphologic parameters were measured: condylar volume (CV), condylar superficial area (CSA), condylar morphological index (CMI), fossa volume (FV), fossa superficial area (FSA), fossa morphological index (FMI),the proportion of the condylar volume in the articular fossa (CV%), the proportion of the condylar superficial area in the articular fossa (CSA%), superior joint space (SJS), anterior joint space (AJS), posterior joint space (PJS), and medial joint space (MJS); by using the 3-matic for Windows (version 11.0, 3-matic Research software, Materialise), Geomagic Wrap for Windows (version 2017 [64 bit], Geomagic Wrap software, Raindrop 3D systems), and Mimics for Windows (version 19.0, Mimics software, Materialise) for reconstructing 3D model.

### Image registration of bilateral 3D condyle model

In order to further understand the disparity of the condyle on both sides of the uPSB patients, the surface-to-surface matching technique was applied to compare and record the specific areas.

**Fig. 5:**
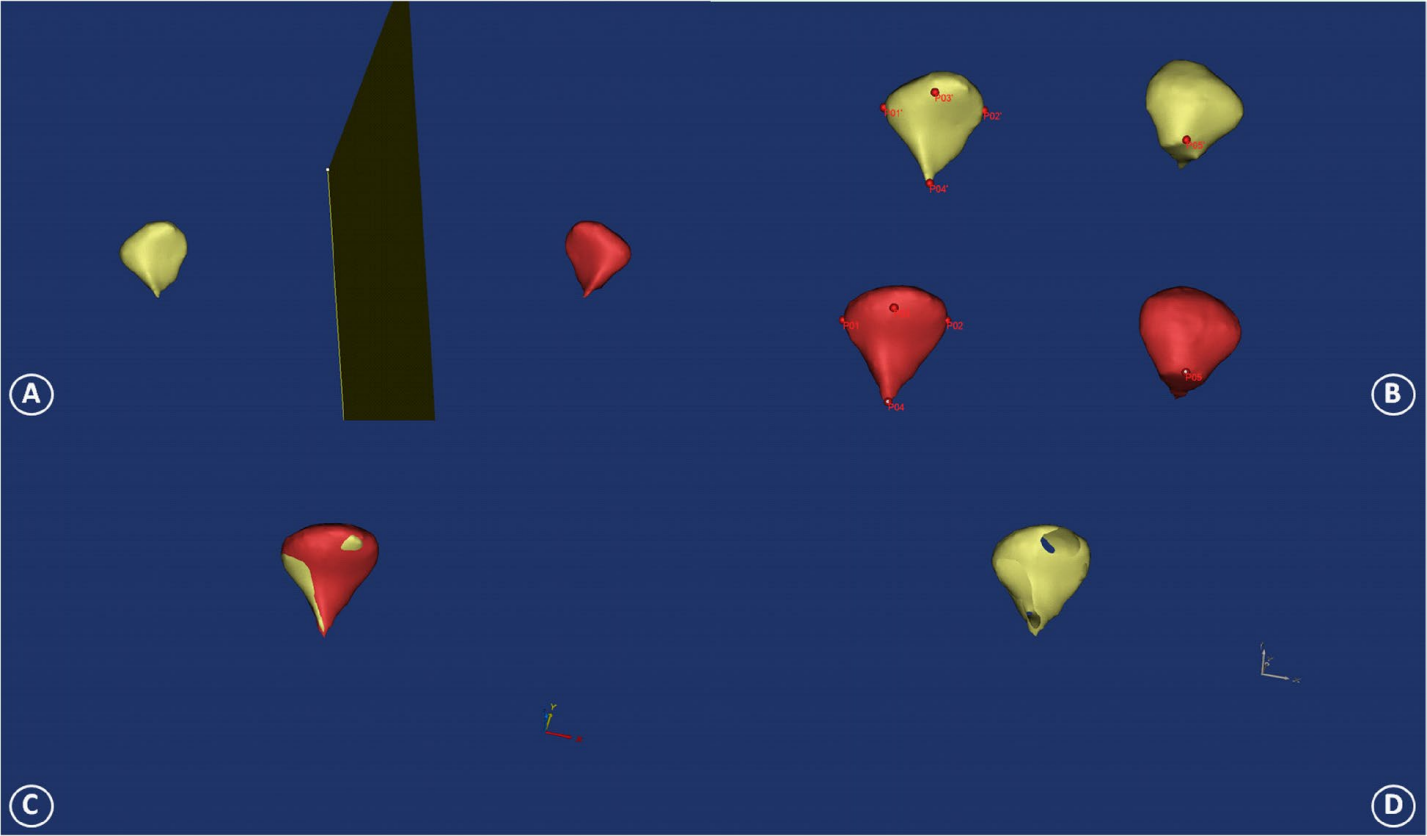
Image registration of bilateral 3D condyle model. **A** Manual segmentation of bilateral 3D reconstructed condyle models. **B** Landmarking five points on 3D condylar model superimposition. **C** and **D** Display of the different sites of bilateral condyles

The preliminary registration of bilateral condyles were carried out by selecting the same 5 points of anatomical landmarks on the surfaces of the 3D models: medial pole of condyle head, lateral pole of condyle head, anterior pole of condyle head, condylar base, sigmoid notch, respectively. To enhance the quality of the superimposition, a surface-based registration was made by using the ‘Best fit alignment’ function. Using the ground truth condylar model of normal side as the reference, the final superimposition and registration were carried out by setting the precision to at least 0.01 mm [16] (Fig. 5A, B).

### Data comparability between healthy individuals and normal side of uPSB patient

To prove the comparability of measured data came from normal side of uPSB patients, a healthy volunteer population with intercuspal occlusion and without any other dentofacial deformities (*n* = 20) was selected to appraise the data distribution and verify its consistency as standard control [17]. If the results have no statistical differences, the data of normal side of uPSB patients have certain representativeness.

### Statistical analysis

Statistical analysis was performed using the Statistical Product and Service Solutions, SPSS (version 26.0, IBM). The Kolmogorov–Smirnov test was used to verify the normality of all data. Normally distributed data are expressed as mean ± standard deviation (SD) ($\overline{x }\pm s$) and are calculated through parameter test. Paired sample *t*-test was used to compare the measurements between uPSB side and normal side [18]; one-way analysis of variance was used to compare the measurements among different age subgroups. Non-normally distributed data are presented as median (first and third quartiles, Q1 and Q3) and are calculated through nonparametric test. Wilcoxon signed-rank sun test was used to compare the measurements between uPSB side and normal side [18]; Kruskal–Wallis *H* test was used to compare the measurements among different age subgroups. The Bonferroni correction was conducted for multiple comparisons. *P* < 0.05 was considered statistically significant.

## Results

### Patients’ general characteristics

**Fig. 6:**
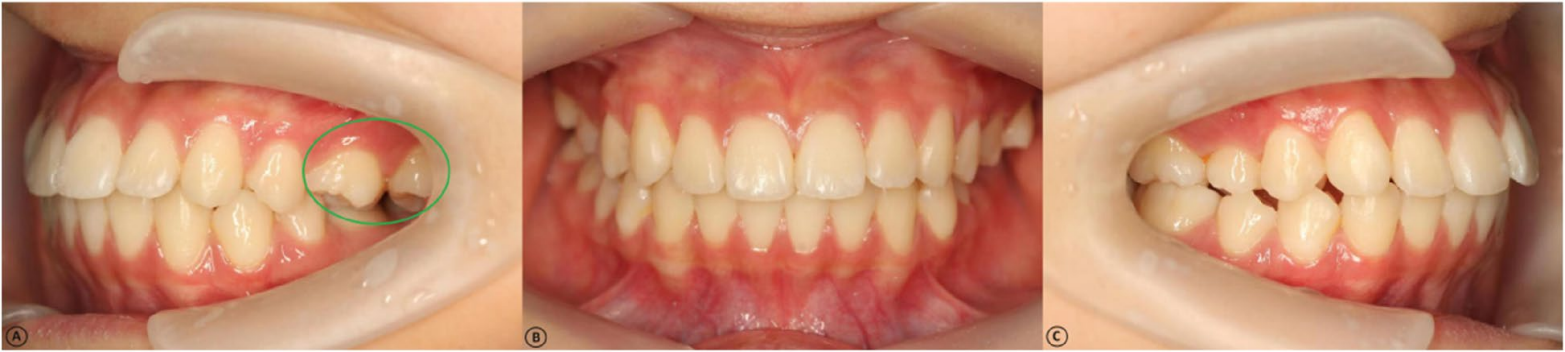
Unilateral posterior scissors-bite malocclusion in the patient population. **A** Left side view, affected tooth position: 26, 27. **B** Frontal view. **C** Right/normal side view

A total of 95 patients (190 joints) met the inclusion criteria. All the patients were affected unilaterally (Fig. 6). Therefore, a self-control grouping (affected and normal side of TMJ) was determined [19]. Patient age ranged from 12 to 54 years (mean ± SD, 24.83 ± 9.45 years), and the female-to-male ratio was 2.96:1 (71 females, 24 males). For the subgroup analysis stratified as 12 ~ 20 years, 21 ~ 30 years, ≥ 31 years, the majority was 21 ~ 30 years (N_②_ = 41), followed by 12 ~ 20 years (N_①_ = 36) and ≥ 31 years (N_③_ = 18).

### TMJ morphologic parameters on scissors-bite side and normal side

The Kolmogorov–Smirnov test result showed that the CV, CSA, CMI, FV, FSA, FMI, CV%, CSA%, AJS, and MJS conformed to the Gaussian distribution (*P* > 0.05); however, the SJS and PJS data were non-normally distributed. The results of the pairwise comparisons between different posterior occlusal states indicated that the CV was significantly larger in the scissors-bite side than in the normal side [(*CV*_S_ = 1740.68 ± 559.80 mm^3^) > (*CV*_N_ = 1662.25 ± 524.88 mm^3^), *P* = 0.027]. The CSA was also significantly larger in the scissors-bite side than in the normal side [(*CSA*_S_ = 818.71 ± 186.82 mm^2^) > (*CSA*_N_ = 792.63 ± 173.44 mm^2^), *P* = 0.030]. The SJS of the scissors-bite side [*SJS*_S_ = 2.46 (*Q1* = 1.61, *Q2* = 3.68) mm] was larger than that of the normal side [*SJS*_N_ = 2.01 (*Q1* = 1.55, *Q2* = 2.87) mm] (*P* = 0.018). The AJS of the scissors-bite side (*AJS*_S_ = 3.94 ± 1.46 mm) was also larger than that of the normal side (*AJS*_N_ = 3.57 ± 1.30 mm) (*P* = 0.017) (Table 1).

**Table 1:** Comparisons of morphologic parameters of TMJ on different occlusal states

Variables	Scissors-bite side	Normal side	*P*_K-S_ value	*t* or *z* value	*P* value
CV (mm^3^)	1740.68 ± 559.80	1662.25 ± 524.88	0.200	2.239	**0.027**
CSA (mm^2^)	818.71 ± 186.82	792.63 ± 173.44	0.200	2.201	**0.030**
CMI	2.08 ± 0.23	2.05 ± 0.23	0.200	1.734	0.086
FV (mm^3^)	597.40 ± 256.52	594.67 ± 252.76	0.200	0.124	0.902
FSA (mm^2^)	586.69 ± 153.35	587.82 ± 149.62	0.200	-0.089	0.929
FMI	0.98 ± 0.23	0.97 ± 0.21	0.200	0.222	0.825
CV%	207.54 ± 135.25	196.87 ± 131.13	0.200	0.973	0.333
CSA%	51.47 ± 36.98	48.17 ± 42.90	0.161	0.836	0.405
SJS (mm)	2.46 (1.61, 3.68)	2.01 (1.55, 2.87)	0.000	-2.376	**0.018**
AJS (mm)	3.94 ± 1.46	3.57 ± 1.30	0.200	-2.426	**0.017**
PJS (mm)	3.03 (2.35, 4.17)	3.07 (2.30, 4.07)	0.015	-0.909	0.363
MJS (mm)	3.37 ± 1.33	3.52 ± 1.62	0.096	-1.100	0.274

*AJS* Anterior joint space, *CV* Condylar volume, *CV%* The proportion of condylar volume in the articular fossa, *CMI* Condylar morphological index, *CSA* Condylar superficial area, *CSA%* The proportion of the condylar superficial area in the articular fossa, *FMI* Fossa morphological index, *FSA* Fossa superficial area, *FV* Fossa volume, *K-S* Kolmogorov–Smirnov, *MJS* Medial joint space, *NADP* Normal articular disc position, *PJS* Posterior joint space, *SJS* Superior joint space

### TMJ morphological parameters between healthy individuals and normal side of uPSB patient

There was no difference within all normal TMJ morphologic parameters came from normal side of uPSB patients, as well as healthy individuals, showing a good consistency [17] (Table 2).

**Table 2:** TMJ morphological parameters between healthy individuals and normal side of uPSB patient

Variables	Healthy individuals (*N* = 20)	uPSB patients (*N* = 20)	*t* or *z* value	*P* value
CV (mm^3^)	1894.61 ± 476.91	1773.26 ± 644.95	-0.648	0.525
CSA (mm^2^)	881.25 ± 158.21	833.88 ± 219.54	0.725	0.477
CMI	2.12 ± 0 .21	2.07 ± 0.24	-0.705	0.489
FV (mm^3^)	477.01 (384.95, 673.03)	637.95 (421.52,740.66)	-0.896	0.370
FSA (mm^2^)	561.18 ± 134.33	574.76 ± 107.27	0.341	0.737
FMI	0.95 ± 0.20	1.00 ± 0.25	0.633	0.534
CV%	156.98 ± 99.70	185.69 ± 116.47	0.979	0.340
CSA%	36.79 ± 21.59	46.50 ± 38.54	1.208	0.242
SJS (mm)	1.90 ± 0.65	1.94 ± 0.98	0.158	0.876
AJS (mm)	2.71 ± 0.99	2.83 ± 1.11	0.296	0.771
PJS (mm)	2.06 (1.63, 3.16)	2.10 (1.82, 2.54)	-0.336	0.737
MJS (mm)	3.82 (2.60, 4.68)	3.40 (3.02, 4.93)	-0.541	0.588

*AJS* Anterior joint space, *CV* Condylar volume, *CV%* The proportion of condylar volume in the articular fossa, *CMI* Condylar morphological index, *CSA* Condylar superficial area, *CSA%* The proportion of the condylar superficial area in the articular fossa, *FMI* Fossa morphological index, *FSA* Fossa superficial area, *FV* Fossa volume, *MJS* Medial joint space, *PJS* Posterior joint space, *SJS* Superior joint space

^*^P < 0.05

^**^P < 0.01

### Subgroup analysis for the comparison of TMJ morphologic parameters of uPSB patients distributed in different ages

The uPSB patients were stratified into three subgroups: *i*. 12 ~ 20 years, *ii*. 21 ~ 30 years, and *iii*. ≥ 31 years. There were statistically significant differences in CV%, CSA%, SJS, AJS and MJS among different age subgroups (*P* < 0.05), whereas the other measuring indicators had no significant difference (Table 3).

**Table 3:** Comparison of TMJ morphologic parameters of uPSB patients distributed in different age subgroups

Variables	Age distribution (years)			*F* or *H* value	*P* value
	**12 ~ 20, (** ***N*** ** = 36)**	**21 ~ 30, (** ***N*** ** = 41)**	** ≥ 31, (** ***N*** ** = 18)**		
**CV (mm**^**3**^**)**					
** uPSB side**	1706.48 ± 644.29	1783.84 ± 491.94	1710.76 ± 548.43	0.211	0.810
** normal side**	1606.76 ± 562.27	1681.79 ± 494.95	1728.74 ± 532.82	0.369	0.692
**CSA (mm**^**2**^**)**					
** uPSB side**	774.44 (627.86, 1013.93)	857.34 (704.30, 935.10)	774.66 (685.32, 941.65)	0.809	0.667
** normal side**	776.44 ± 191.70	797.87 ± 157.03	813.08 ± 177.71	0.296	0.744
**CMI**					
** uPSB side**	2.05 ± 0.25	2.10 ± 0.23	2.09 ± 0.18	0.560	0.573
** normal side**	2.02 ± 0.24	2.07 ± 0.23	2.09 ± 0.22	0.757	0.472
**FV (mm**^**3**^**)**					
** uPSB side**	653.87 ± 233.60	567.39 ± 271.89	552.81 ± 257.92	1.438	0.243
** normal side**	660.82 ± 283.05	551.73 ± 216.62	560.19 ± 250.85	2.036	0.136
**FSA (mm**^**2**^**)**					
** uPSB side**	618.79 ± 136.96	564.12 ± 165.93	573.93 ± 151.84	1.303	0.277
** normal side**	621.59 ± 151.58	564.08 ± 139.52	574.38 ± 163.44	1.522	0.224
**FMI**					
** uPSB side**	1.03 ± 0.22	0.95 ± 0.24	0.93 ± 0.21	1.636	0.200
** normal side**	1.03 ± 0.23	0.95 ± 0.19	0.93 ± 0.24	1.720	0.185
**CV%**					
** uPSB side**	258.67 ± 136.09	173.79 ± 134.61	182.16 ± 106.84	4.474	0.014*
** normal side**	245.28 (144.39, 315.25)	132.44 (63.49, 224.39)	182.69 (86.51, 247.43)	13.196	0.001*
**CSA%**					
** uPSB side**	62.23 ± 44.44	45.45 ± 31.31	43.63 ± 28.10	2.555	0.038*
** normal side**	64.10 (35.07, 81.42)	32.94 (21.14, 61.21)	46.97 (16.67, 66.96)	7.636	0.022*
**SJS (mm)**					
** uPSB side**	1.91 (1.40, 3.12)	2.63 (2.02, 4.22)	2.36 (1.59, 4.27)	8.954	0.011*
** normal side**	1.77 (1.10, 2.73)	2.07 (1.77, 3.15)	2.46 (1.47, 4.10)	6.207	0.045*
**AJS (mm)**					
** uPSB side**	3.62 ± 1.37	4.14 ± 1.43	4.14 ± 1.66	1.444	0.241
** normal side**	3.36 ± 1.19	3.40 ± 1.25	4.50 ± 1.47	5.680	0.005*
**PJS (mm)**					
** uPSB side**	3.07 (2.42, 4.10)	3.15 (2.28, 4.42)	2.62 (1.74, 3.63)	1.880	0.391
** normal side**	2.96 (1.96,3.96)	3.08 (2.50, 4.42)	3.43 (2.11, 4.78)	2.827	0.243
**MJS (mm)**					
** uPSB side**	3.12 ± 0.99	3.47 ± 1.47	3.61 ± 1.59	1.045	0.356
** normal side**	2.82 (1.67, 3.49)	3.59 (2.64, 4.48)	4.18 (2.96, 5.76)	8.274	0.016*

*AJS* Anterior joint space, *CV* Condylar volume, *CV%* The proportion of condylar volume in the articular fossa, *CMI* Condylar morphological index, *CSA* Condylar superficial area, *CSA%* The proportion of the condylar superficial area in the articular fossa, *FMI* Fossa morphological index, *FSA* Fossa superficial area, *FV* Fossa volume, *MJS* Medial joint space, *PJS* Posterior joint space, *SJS* Superior joint space

^*^considered as significantly statistical difference

### Deviation analysis and assessment of condylar registration of 3D models

Once the deviation documentation was carried out (Fig. 5C, D), the percentages (%) of condylar differences between the uPSB side and normal side of all subjects were calculated as 23% (posterior slope), 21% (condylar apex), 20% (anterior slope), 19% (lateral surface), 17% (medial surface), in that order.

## Discussion

A buccal cross-bite with a whole segment of the upper teeth outside the lower arch is particularly termed as scissors-bite. Scissors-bite, with an estimated occurrence of 1.5% in the general population, is even rarely observed during the primary dentition period [20]. Nevertheless, such reported prevalence may be underestimated because an afflicted individual is often unaware that possessing a scissors-bite. This condition not only remains a clinical challenge for orthodontists, but also brings the maxillofacial surgeons’ attention due to its indirect repercussions for TMJ physiological behavior.

The aetiology of TMJ lesion is multifactorial that associated with many initiating, predisposing, and perpetuating factors [21]. Despite several types of occlusal discrepancies have been considered as variable features of the norm, it has been controversial whether malocclusion can cause alterations in the structure of TMJ. On the one hand, occlusal disorders or/and bad habitual mastication are sometimes indicated to the own weakness of TMJ; the TMJ-occlusion couple is often symbiotic, on the other hand, developing together in relation to its interplay [22]. Prolongation of posterior crossbite can cause permanent changes in bony support, and possibly in the growth center at the TMJ, showing that malocclusions, especially transverse anomalies, have a marked effect on mandibular condyle morphology [23, 24]. Patients with bilateral posterior crossbite have asymmetrical condyles that might be at risk for the development of future skeletal mandibular asymmetries [25–28].

Posterior scissors-bite is also a kind of asymmetric occlusal state. In this present study, for the first time, we compared the condylar morphologic variables correlated with fossa between uPSB side and contralateral normal side based on 3D model reconstructed by CBCT images. The data herein provided can contribute to the understanding of what the osseous changes of TMJ can be presented in response to malocclusal traits, in uPSB patients. In addition, our discoveries suggest that the change in trend of condylar morphology in the uPSB malocclusion side associates with different ages.

To testify the rationality of the data source collected, we exclusively performed the comparisons between the healthy individuals and normal side of uPSB patient. No differences in the morphologic variables proved their data distribution had a good consistency [17]. Our study also showed statistically significant differences in CV, CSA, SJS, and AJS values when comparing the “cases” (affected side) and “controls” (normal side) from one uPSB patient, which explained that this asymmetric occlusal state may lead to pathological changes in the shape and position of the condyle. Abnormal condylar morphology can additionally bring about anomalies of the relative position between the condyle and articular disk, which will bring about anterior disk displacement of the TMJ and is consistent with the clinical symptoms of TMJ internal derangement [17, 19]. Another point to consider is that different duration of this disease may have different impact on the shape and position of the condyle, owing that the condylar adaptive remodeling is a continuous and dynamic process. Rodrigues et al. [29] reported that the stress acting on the bilateral condyles was relatively balanced, so the dimensional and positional remodeling between the right and left condyles in subjects with Class I malocclusion appeared to be symmetrical. This indirectly suggested that asymmetric occlusion could lead to asymmetry of bilateral condyles, which also supported our research results.

Unlike most other joints in the human body, the articular surfaces of which are covered by hyaline cartilage, in the TMJ the articular surfaces are covered by fibrocartilaginous tissue. There is a locus of hyaline cartilage within the condylar head, however, that serves as a major mandibular growth center rather than a stress bearing surface. More importantly, as the growth center of the mandible, the condyle is in the process of rebuilding all its life. It is noteworthy that the growth and development of condyle lag behind the onset of occlusion. Both dentition and TMJ are easily affected by occlusive factors, although the self-remodeling ability of TMJ is stronger than that of dentition. The results of subgroup analysis of age distribution in this present study indicated that CV%, CSA%, SJS, AJS, and MJS values showed statistically significant differences among three age subgroups (*i*. 12 ~ 20 years, *ii*. 21 ~ 30 years, and *iii*. ≥ 31 years), which indicating different uPSB span accompanied by varying degrees of degenerative alteration of the condyle. The tendency of SJS, AJS, and MJS showed the position of condyle shifted backward, outward and downward along with getting aged. Hence, the orthodontic intervention should be carried out as soon as possible to avoid causing abnormal condylar morphology and even TMJ asymmetry.

To date, the relationship between occlusal interference and TMD is still a very disputable problem, although the results of this study discovered a certain trend toward making a weak correlation between condylar position and asymmetry and unilateral posterior scissors-bite. Moreover, because this feasibility study contained a small as well as mismatched sample size in three different age subgroups, the findings should be verified by studies involving proportionately larger sample sizes. In addition, the sample size of women was larger than that of men, reflecting the higher prevalence of uPSB malocclusion in women [6, 30], accordingly, lack of sex matching should be overcome to clarify the potential correlation between these TMJ parameters and sex distribution.

## Conclusion

Regarding as an asymmetric occlusal state, unilateral posterior scissors-bite malocclusion resulted in the changes of condylar morphology, and further affected the relative position between condyloid process and glenoid fossa, and thus finally made against the TMJ function. Adolescent and adult uPSB patients had asymmetrical condyles between the right and left side, presenting different morphology (CV and CSA) and different position (SJS and AJS). Furthermore, the varying sites of bilateral condyles were focused on posterior slope (23%), condylar apex (21%), anterior slope (20%), lateral surface (19%), and medial surface (17%), respectively. Next investigation concerning the evaluation of condylar and ramal vertical asymmetry in patients with unilateral and bilateral posterior scissors-bite malocclusion using 3D reconstructive imaging technique is performing in our succeeding work.

## Data Availability

The analyzed data sets generated during the study are available from the corresponding author on reasonable request.
